# Biomimetic nanomaterial-facilitated oxygen generation strategies for enhancing tumour treatment outcomes

**DOI:** 10.3389/fbioe.2022.1007960

**Published:** 2022-10-05

**Authors:** Zhongwen Yang, Changsong Shi, Dongliang Cheng, Yu Wang, Yan Xing, Fanfan Du, Fangfang Wu, Yao Jin, Yueli Dong, Mengli Li

**Affiliations:** Department of Pediatric, Henan Provincial People’s Hospital, Zhengzhou, China

**Keywords:** tumour hypoxia, nanomaterials, biomimetics, tumour microenvironment, tumour treatment

## Abstract

Hypoxia, as a typical hallmark of the tumour microenvironment (TME), has been verified to exist in most malignancies and greatly hinders the outcome of tumour treatments, including chemotherapy, photodynamic therapy, radiotherapy, and immunotherapy. Various approaches to alleviate tumour hypoxia have been reported. Among them, biomimetic nanomaterial-facilitated tumour oxygenation strategies, based on the engagement of human endogenous proteins, red blood cells, the cell membrane, and catalase, are the most impressive due to their excellent tumour active-targeting ability and superior tumour-selective capability, which, however, have not yet been systematically reviewed. Herein, we are ready to describe the current progress in biomimetic nanomaterial-facilitated tumour oxygenation strategies and corresponding improvements in tumour treatment outputs. In this review, the underlying mechanism behind the superior effect of these biomimetic nanomaterials, compared with other materials, on alleviating the hypoxic TME is highlighted. Additionally, the ongoing problems and potential solutions are also discussed.

## Introduction

Tumour hypoxia, which is usually characterized mainly by insufficient oxygen supply and caused by the rapid proliferation of tumour cells and incomplete development of the vascular system, has been shown to exist in most solid tumors (Brahimi-Horn et al., 2007; Chu et al., 2017; Huo et al., 2020; Yang et al., 2022a). Moreover, tumour hypoxia, as a typical hallmark of the tumour microenvironment (TME), has been recently proven to be one of the main reasons for cancer treatment failure (Carlson et al., 2011; Bouleftour et al., 2021). The hypoxic TME seriously reduces the sensitivity of tumours to conventional chemotherapy, radiotherapy (RT), and photodynamic therapy (PDT) (Baggiani et al., 2011; Baumann et al., 2016; Ayob and Ramasamy, 2021; Bouleftour et al., 2021; Carvalho et al., 2021). Meanwhile, the hypoxic TME also induces an immunosuppressive TME through four aspects: 1) initiating macrophage polarization from M1-phenotype (anti-tumour) to M2-phenotype (pro-tumour) (Delprat et al., 2020; Lee et al., 2020; Wu et al., 2020); 2) upregulating the expression of immune checkpoint blockades, such as programmed death ligand 1 (PD-L1) and programmed death 1 (PD-1) (Noman et al., 2014; Voron et al., 2015); 3) recruiting the immunosuppressive cells, (M2-phenotype macrophages, regulatory T cells, and myeloid-derived suppressor cells) (Huang et al., 2012; Lindau et al., 2013); and 4) preventing dendritic cells from maturing (Gabrilovich et al., 1996). The hypoxia-associated TME not only interferes with T lymphocyte activation and proliferation but also induces rapid tumour proliferation and metastasis. As such, reversing the hypoxic TME would be an effective strategy for enhancing tumour treatment outcomes.

Some approaches to alleviate tumour hypoxia, such as mitochondrial respiration inhibition, tumour vasculature, and oxygen (O_2_) delivery, have been extensively reported. While these strategies indeed have the ability to alleviate tumour hypoxia to some extent, they also lead to inescapable side effects on normal tissues due to their nontargeting and nonselectivity toward tumour cells (Zhou et al., 2018; Song et al., 2020; Tang et al., 2020; Liu et al., 2021a; Yang et al., 2022b). Some small molecules were also reported that could effectively carry or generate oxygen *in vitro*. However, their short blood half-life and poor tumor accessibility greatly limit the tumor oxygenation efficiency *in vivo*. Recently, biomimetic nanomaterial-facilitated tumour oxygenation strategies have been reported to be capable of making up for the shortcomings of the abovementioned tumour oxygenation strategies (Chen et al., 2019; Feng et al., 2019; Dong et al., 2020; Fang et al., 2021; Chen et al., 2022). For example, when hypoxia regulators (*e.g.,* mitochondrial respiration inhibitors, tumour vasculature antagonists, and O_2_ carriers) are encased by human endogenous proteins, erythrocytes, red blood membranes, platelet membranes, cancer cell membranes, or exosomes, their tumour accessibility and blood half-life can be greatly improved (Gao et al., 2018; Gao et al., 2020; Gong et al., 2021; Gong et al., 2022). Furthermore, natural catalase (CAT) and nanozymes with CAT-like properties can also overcome the hypoxic TME through the *in situ* catalytic decomposition of tumour-overexpressed hydrogen peroxide (H_2_O_2_) and the *in situ* generation of O_2_ (Liang et al., 2017; Ai et al., 2018; Liang et al., 2018; Chang et al., 2019; Liu et al., 2019; Yu et al., 2019).

In this review, we summarize the present advances in biomimetic nanomaterial-empowered tumour oxygenation strategies. These biomimetic nanomaterials integrate multitudinous advantages, such as excellent biocompatibility, prolonged circulation time, immune evasion, and tumour-targeting and tumour-selecting efficacy, which explains the superior effect of these biomimetic nanomaterials on alleviating the hypoxic TME and improving tumour treatment outputs compared with other materials (Kuo et al., 2014; Jiang et al., 2019a; Li et al., 2020a; Li et al., 2021a). In the present review, not only are the merits highlighted, but the ongoing problems and the potential solutions are also discussed and concluded. We hope this review will help researchers better understand the field of tumour hypoxia, which will promote the development of cancer treatment.

## Human endogenous protein-facilitated tumour oxygenation strategies

Human endogenous proteins, such as haemoglobin (Hb), albumin (HSA), glutathione S-transferase (GST), and bovine serum albumin (BSA), have long been employed to fabricate biomimetic nanomaterials through an eco-friendly biomimetic synthesis technology due to their wonderful biocompatibility, good flexibility, and green synthesis features (Zhang et al., 2012; Yang et al., 2016a; Yang et al., 2017; Zhang and Han, 2018). Recently, human endogenous protein-fabricated biomimetic nanomaterials have also been reported to alleviate tumour hypoxia and improve tumour treatment outcomes (Yang et al., 2016b; Zhao et al., 2017; Yang et al., 2018).

### Haemoglobin -based biomimetic nanomaterials

**FIGURE 1:**
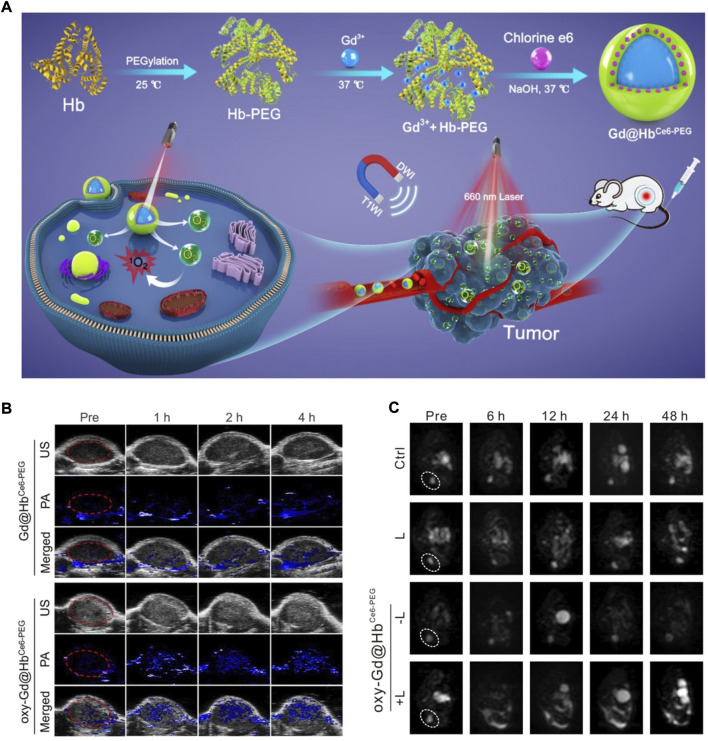
**(A)** A schematic diagram showing the Hb-mediated biomimetic nanomaterials (Gd@HbCe6-PEG NPs) for tumor oxygenation. **(B)** Representative PA images of the tumor sites after indicated treatments. **(C)** Representative DW images of the tumor sites after indicated treatments. Reprocessed with permission from ref 52. Copyright 2020, Ivyspring International Publisher.

Hb is a special protein that has an iron-containing haem group (Zhao et al., 2016). The iron state of Hb can be transformed from Fe^2+^ (ferrous) to Fe^3+^ (ferric) under high O_2_ pressure (oxidative environment), allowing for efficient O_2_ binding and transport (Zhu et al., 2019). However, under low O_2_ pressure (reductive environment), the iron state returns to Fe^2+^, which is responsible for the rapid O_2_-unleashing capacity. By virtue of their superior O_2_-carrying/release capability, Hb-based biomimetic nanomaterials have been applied to tumour oxygenation to boost antitumor therapy. Recently, Zhang’s group designed Hb-mediated biomimetic nanomaterials (Gd@HbCe_6_-PEG NPs) to overcome the hypoxic TME-limited efficacy of PDT (Figure 1A) (Shi et al., 2020). The paramagnetic O_2_-evolving Gd@HbCe_6_-PEG NPs were synthesized through an eco-friendly biomimetic synthesis technology based on Hb’s biological template role. The loading of Ce_6_ was mainly due to hydrophobic interactions, while the loading of Gd^3+^ metal ions occurred *via* the strong affinity of transition metals with the amino acid residues of Hb. PEGylation endowed biomimetic nanomaterials with better biocompatibility and stability. They next assessed the oxygen delivery capability of oxy-Gd@HbCe_6_-PEG by measuring oxygenated haemoglobin (HbO_2_) through PA imaging. HbO_2_, as a positive indicator of tumour oxygenation, displayed an increased signal intensity over time after the i.v. injection of oxy-Gd@HbCe_6_-PEG NPs (Figure 1B), suggesting that Gd@HbCe_6_-PEG is an applicable O_2_ nanocarrier. Diffusion-weighted (DW) imaging *in vivo* is a powerful technology for measuring the direction and extent of the localized diffusion of water molecules. As a functional magnetic resonance (MR) imaging modality, DW imaging can reflect visible changes in tumour size and morphology. According to Figure 1C, the lowest intensity on the DW imaging map of the lesion areas was shown in the oxy-Gd@HbCe_6_-PEG NP-rendered PDT group, indicating a noticeable treatment outcome.

### HSA-based biomimetic nanomaterials

**FIGURE 2:**
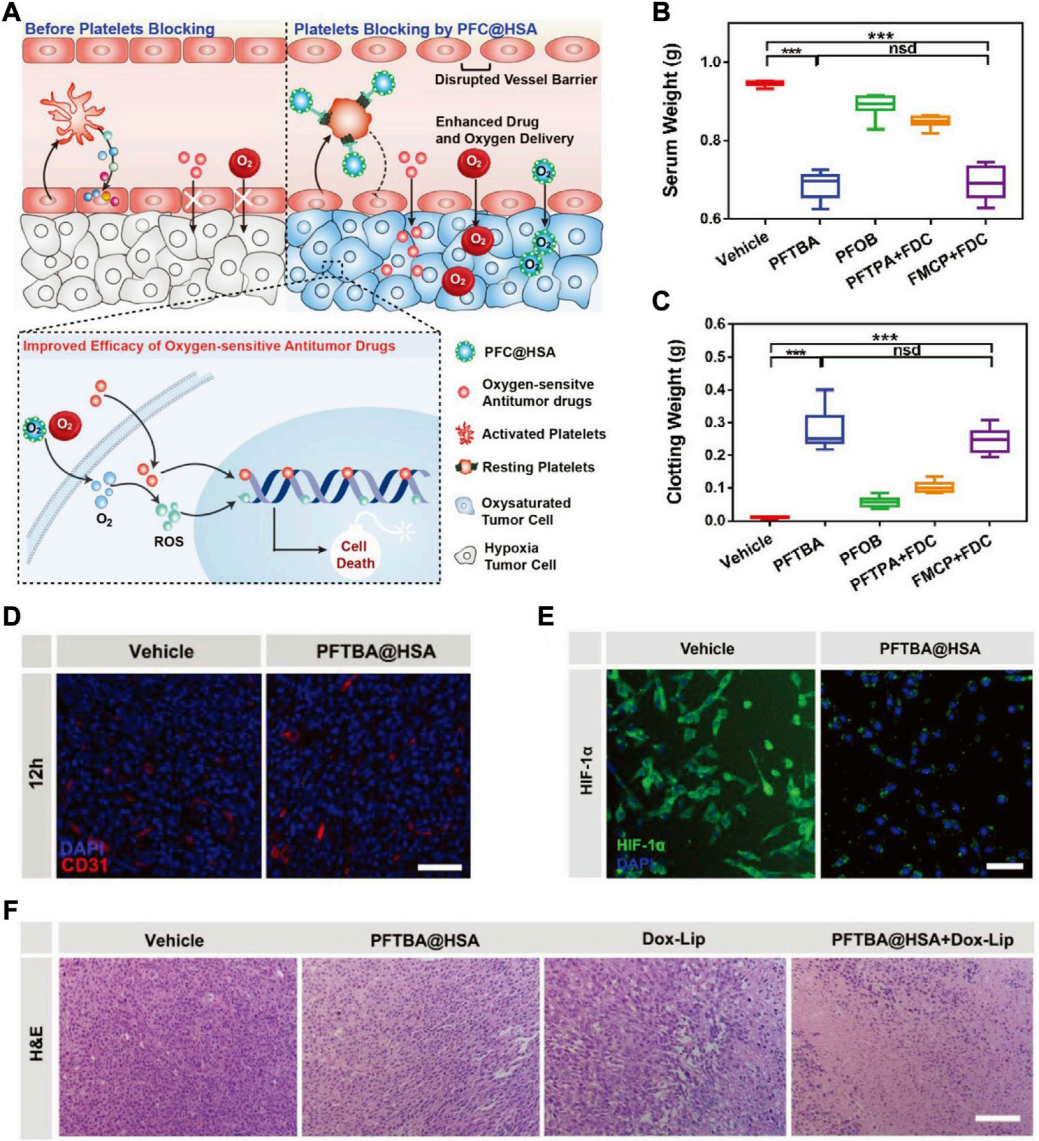
**(A)** The scheme showing PFTBA@HSA mediated platelet blocking for improved efficacy of oxygen-sensitive antitumor drugs. **(B,C)** Quantification of serum weight **(B)** and clotting weight **(C)**. **(D)** Representative immunofluorescence images of CD31 staining to depict tumor vessels (scale bar = 50 μm). **(E)** Representative immunofluorescence images of HIF-1α staining to track the degree of tumor hypoxia. **(F)** Representative images of H&E staining of the tumor tissues after indicated treatments (scale bar = 100 μm). Statistical significance was calculated by two-tail Student’s t-test. *p < 0.05; **p < 0.01; ***p < 0.001. Reprocessed with permission from ref 53. Copyright 2018, WILEY-VCH Verlag GmbH & Co. KGaA, Weinheim.

HSA is the protein in human plasma, accounting for approximately 60% of the total plasma protein. Recently, Wu’s group selected HSA as the drug carrier to construct PFTBA@HSA nanoparticles (PFTBA@HSA NPs) (Zhou et al., 2018). The constructed HSA-mediated biomimetic nanomaterials could overcome the platelet-maintained tumour blood barrier and improve oxygen-sensitive antitumor efficacy (Figure 2A). A clot retraction experiment was performed to analyse the effects of PFTBA@HSA NPs on platelet functions. A shrinking amount of released serum that matched the inhibited shrinkage of blood clots was observed after treatment with PFTBA@HSA NPs (Figures 2B,C), suggesting the blocking platelet function of PFTBA@HSA NPs. After effective platelet inhibition by PFTBA@HSA NPs, the tumour vessel barriers were successfully disrupted along with an enhanced tumour vessel permeability (Figure 2D) and a decreased degree of tumour hypoxia (Figure 2E). Eventually, the strong tumour oxygen perfusion assisted by PFTBA@HSA NP treatment amplified ROS production and cell death (Figure 2F).

## Red blood cell-facilitated tumour oxygenation strategies

The principal function of red blood cells (RBCs) is delivering oxygen to all tissues in the body, including tumors (Beutler et al., 1977). Inspired by this, some RBC-based strategies to overcome hypoxia have been designed and release oxygen under hypoxic conditions (Tang et al., 2016).

**FIGURE 3:**
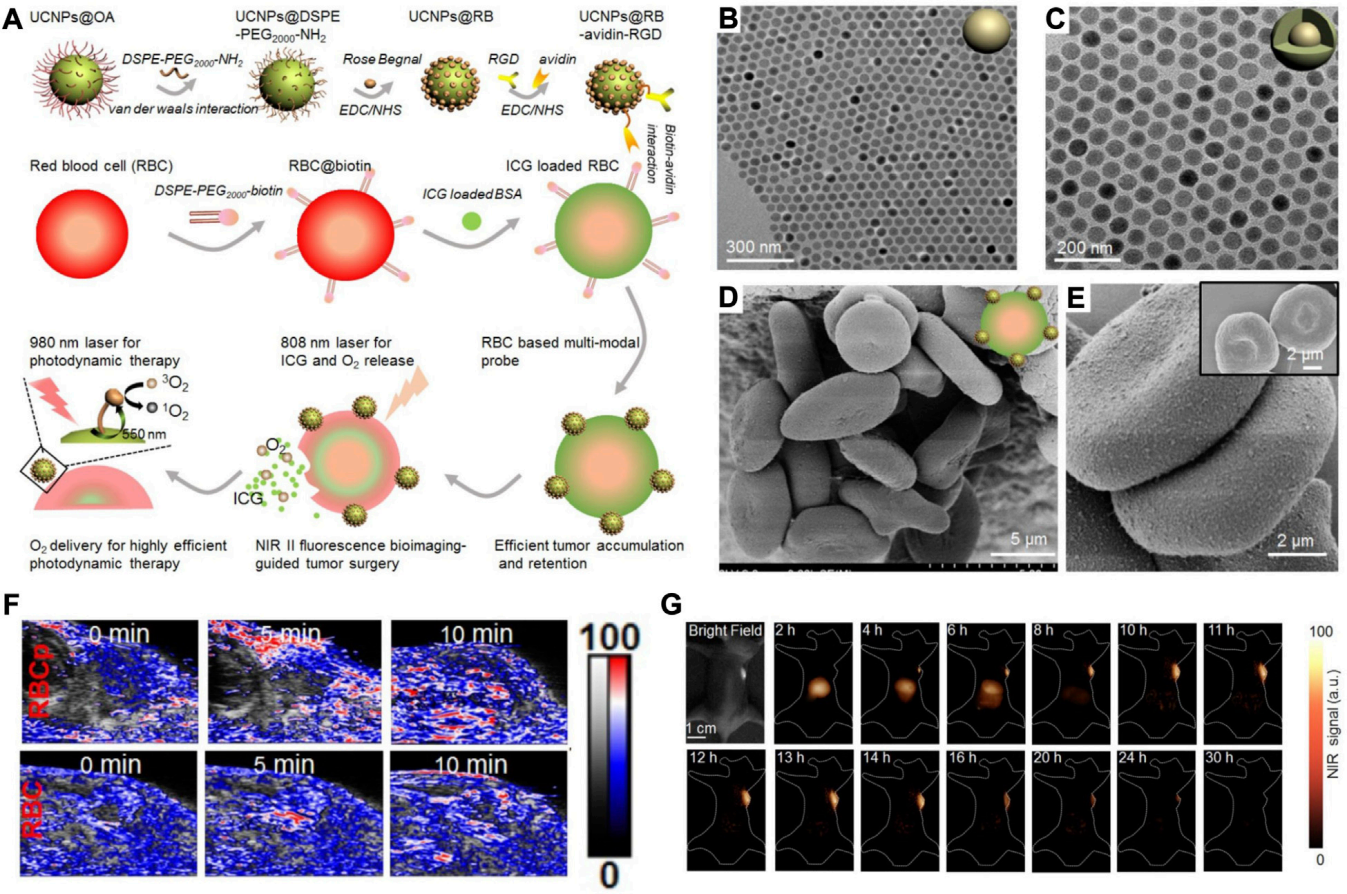
**(A)** The scheme showing the fabrication of RBCp and the application for NIR II fluorescence bioimaging-guided tumor surgery and PDT. **(B,C)** TEM images of NaGdF4:Yb, Er **(B)** and NaGdF4:Yb,Er@NaGdF4 **(C)**. **(D,E)** SEM images of RBC-based multimodal probes attached without UCNPs **(D)** and with UCNPs **(E)**. **(F)** Representative PA images of tumors after indicated treatments. **(G)** NIR II fluorescence bioimaging of the tumor-bearing mice after the *i.v.* injection of RBCp. Reprocessed with permission from ref 56. Copyright 2019, Ivyspring International Publisher.

As haemoglobin is bound to oxygen (oxyhemoglobin) and surrounded by the phospholipid bilayer of RBCs, bursting the RBS membranes can release oxygen to overcome hypoxia in the tumour. Due to its high spatial and temporal precision, laser light is a widely studied remote trigger in the drug release process. To achieve better controlled release outcomes, the Food and Drug Administration (FDA)-approved agent indocyanine green (ICG) was used. Based on this, an RBC-based biomimetic nanomaterial (UCNPs@RB@RGD@avidin crosslinked with RBC@ICG@biotin) was fabricated for near-infrared (NIR) II fluorescence bioimaging-guided tumour surgery and tumour oxygenation-boosted PDT (Figure 3A) (Wang et al., 2019). In this RBC-based biomimetic nanomaterial, UCNPs (lanthanide-doped NaGdF_4_:Yb,Er@NaGdF_4_ nanocrystals) were synthesized for translating 980 nm laser irradiation into 550 nm luminescence and demonstrated a core-shell nanostructure (Figures 3B,C). Additionally, the SEM and TEM results in Figures 3D,E show that lanthanide-doped NaGdF_4_:Yb,Er@NaGdF_4_ nanocrystals were successfully anchored onto the membranes of RBCs without any alteration of the RBC morphology. The *in vivo* results demonstrate that the RBCp structures could be completely destroyed, and O_2_ was released immediately and activated by 808 nm laser irradiation for 10 min (Figure 3F). Moreover, the RBCps could also be used in NIR II fluorescence bioimaging-guided liver tumour surgery, as NIR II fluorescence signals can be observed within the liver tumour for 4 h (Figure 3G). The results showed that the modified RBCs could absorb heat and swell, resulting in the burst release of ICG and O_2_, which could overcome hypoxia in tumours and amplify PDT output.

**FIGURE 4:**
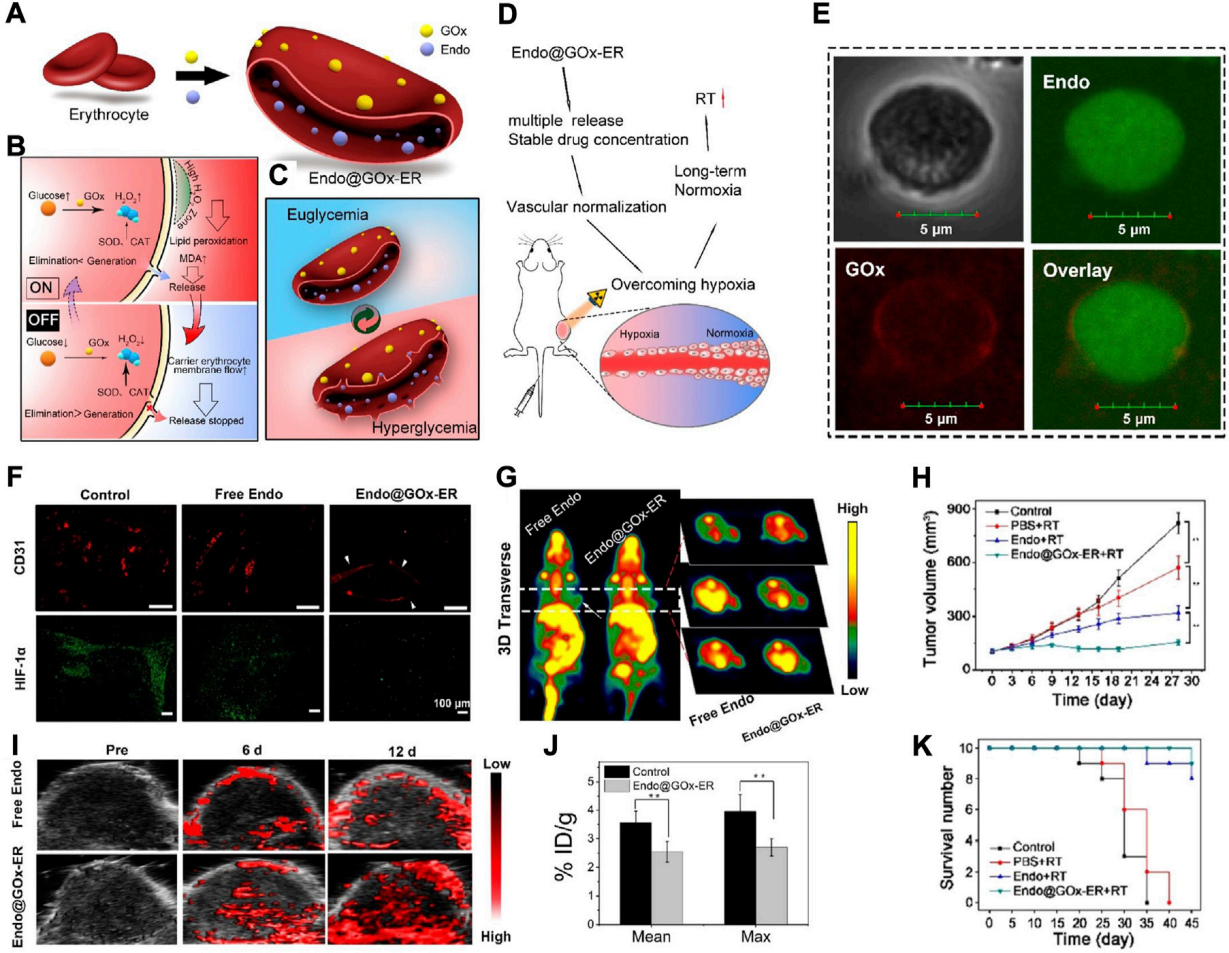
**(A)** The scheme showing the synthesis of Endo@GOx-ER. **(B,C)** The scheme displaying glucose-mediated release of Endo from Endo@GOx-ER. **(D)** The scheme representing the released Endo is capable of promoting tumor vascular normalization, reversing tumor hypoxia, and improving therapeutic outcomes of RT. **(E)** Confocal images demonstrating the encasement of Endo by biotin-GOx. The scale bar is 5 μm. **(F)** Immunofluorescence analysis of tumor sections stained with CD31 and HIF-1α. The scale bar is 100 μm. **(G)** Representative PA images showing the ratio of oxygenated hemoglobin to deoxygenated hemoglobin in tumors after indicated treatments. **(H)** Representative micro-PET scan images showing the absorption of ^18^F-MISO in tumors after indicated treatments. **(I)** % ID/g of ^18^F-MISO after indicated treatments. **(J,K)** Tumor volume **(J)** and survival number **(K)** of mice after indicated treatments. Reprocessed with permission from ref 59. Copyright 2020, American Chemical Society.

Antiangiogenic drugs (such as Endo) have been demonstrated to be able to alter the oxygenation status of a tumour, but most of them fail to acquire satisfactory clinical therapeutic effects, mainly due to their unstoppable degradation and unstable features *in vivo* (Ding et al., 2014; Li et al., 2018). To solve this problem, Xia et al. recently reported a RBC-based biomimetic nanomaterial (Endo@GOx-ER), in which GOx served as a glucose-activated switch for responding to glucose and releasing Endo (Figure 4A) (Huang et al., 2020). Under hyperglycaemia, the accumulation of H_2_O_2_ (the product of GOx-catalysed glucose oxidation) would promote pore formation on RBCms. In normoglycaemia, the Endo release would be suppressed as the pore closed, thus contributing to a pulsatile release manner and sustained high plasma levels (Figures 4B–E). According to the immunofluorescence analysis of tumour sections stained with CD31 and HIF-1α as well as the representative PA images and micro-PET scanning images, it was obvious that Endo@GOx-ER treatment resulted in vascular normalization and accomplished long-term tumour hypoxia relief (Figures 4F–I). When Endo@GOx-ER treatment was combined with RT, persistent tumour regression and higher survival were obtained (Figures 4J,K), suggesting that this RBC-based biomimetic nanomaterial has clinical potential in overcoming tumour hypoxia-limited RT.

## Cell membrane-facilitated tumour oxygenation strategies

To achieve controlled drug release and overcome tumour hypoxia, bionic functional membranes derived from RBC membranes, platelet membranes, tumour cell membranes, etc., are valid choices (Zhang et al., 2018; Zhang et al., 2019a; Feng et al., 2019; Li et al., 2020a; Lyu et al., 2021).

### Red blood cell membrane-based biomimetic nanomaterials

The possibility of using RBC membranes, termed RBCms, as drug carriers has become popular (Meng et al., 2021). Considering the potential clinical usefulness, RBCms are readily available, cost-effective, and allow scaled up preparation. Their long half-life (approximately 28 days) and low immunogenicity in circulation make them particularly well-suited to serve as a popular coating for chemotherapy drugs (Li et al., 2020a).

**FIGURE 5:**
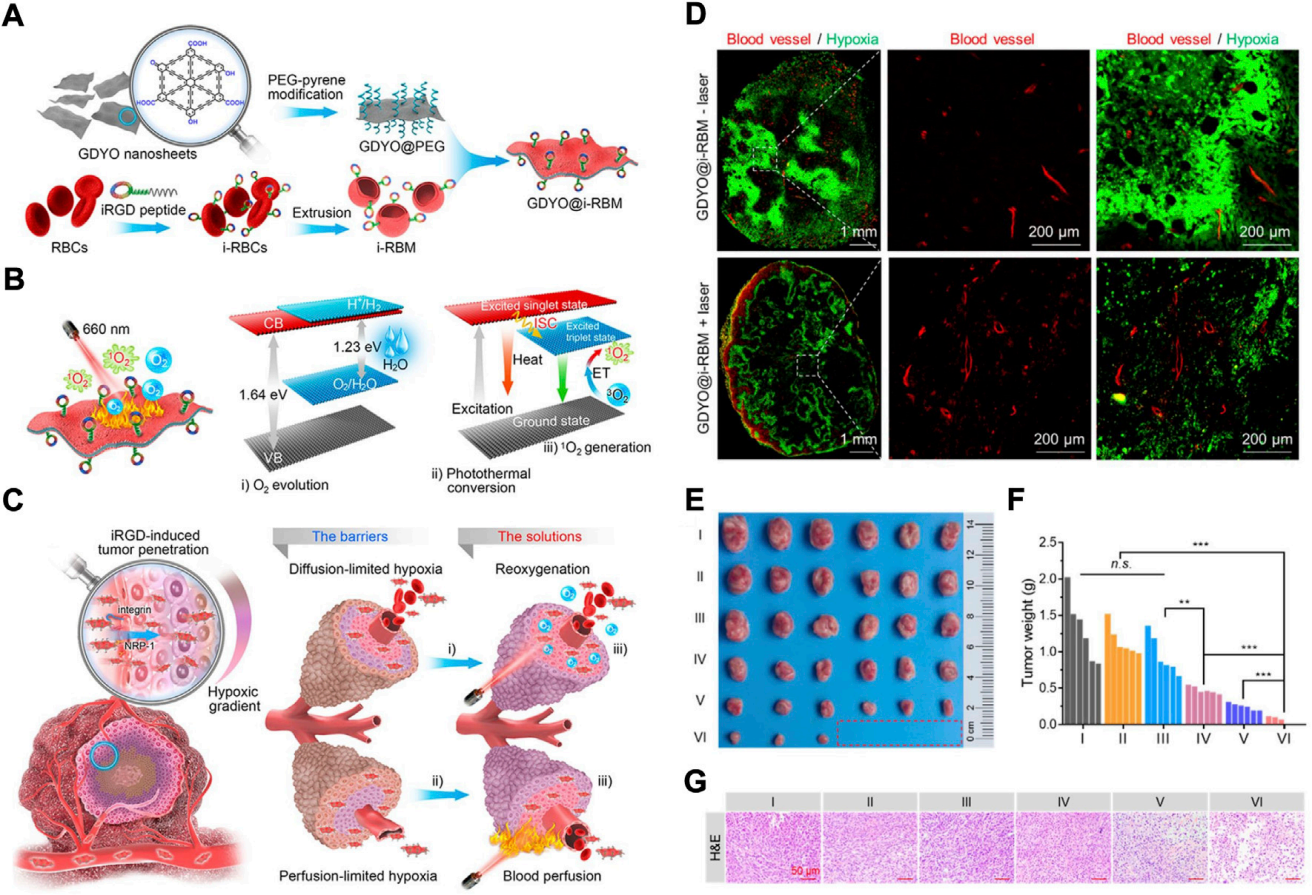
**(A)** The Scheme showing the synthetic process of GDYO@i-RBM. **(B)** The Scheme showing the working principles of GDYO@i-RBM: **(C)** The Scheme showing the reoxygenation process. **(D)** Immunofluorescence analysis of tumor sections stained with anti-CD31 antibody and anti-pimonidazole antibody after indicated treatments. **(E–G)** Photos **(E)**; tumor weight **(F)** and representative images of H&E staining **(G)** of the tumor tissues after I**–VI** treatments (I: PBS-laser; II: PBS + laser; III: GDYO@i-RBM-laser; IV: GDYO@PEG + laser; V: GDYO@RBM + laser; VI: GDYO@i-RBM + laser). Statistical significance was calculated by two-tail Student’s t-test. *p < 0.05; **p < 0.01; ***p < 0.001. Reprocessed with permission from ref 64. Copyright, 2019 American Chemical Society.

As we know RBCs coursing through veins primarily transport oxygen throughout the body, including the tumour. This has inspired the utilization of RBCms as oxygen carriers to overcome both diffusion-limited and perfusion-limited hypoxia (Figure 5) (Jiang et al., 2019b). In detail, a functional iRGD peptide was modified on the RBCms to enhance extravascular and hypoxic region penetration. The graphdiyne oxide (GDYO) nanosheets were loaded into iRGD-RBCm and denoted GDYO@i-RBM (Figure 5A **)**. GDYO@i-RBM showed prolonged blood circulation and enlarged extravascular and hypoxic region penetration because of the functional iRGD peptide. When exposed to irradiation with a 660 nm laser, GDYO nanosheets can evolve sufficient O_2_ to relieve perfusion-limited hypoxia; at the same time, the hyperthermia effect of GDYO overcomes perfusion-limited hypoxia because of the dilation of vessels and blood perfusion (Figure 5B). After i.v. injection, GDYO@i-RBM synchronously alleviated diffusion- and perfusion-limited hypoxia (Figure 5C **)** and further enhanced PDT, resulting from the changes in tumour volume and weight and pathological changes **(** Figures 5E–G **)**. This work sheds new light on RBCm-based biomimetic nanomaterials for overcoming hypoxia.

### Platelet membrane-based biomimetic nanomaterials

**FIGURE 6:**
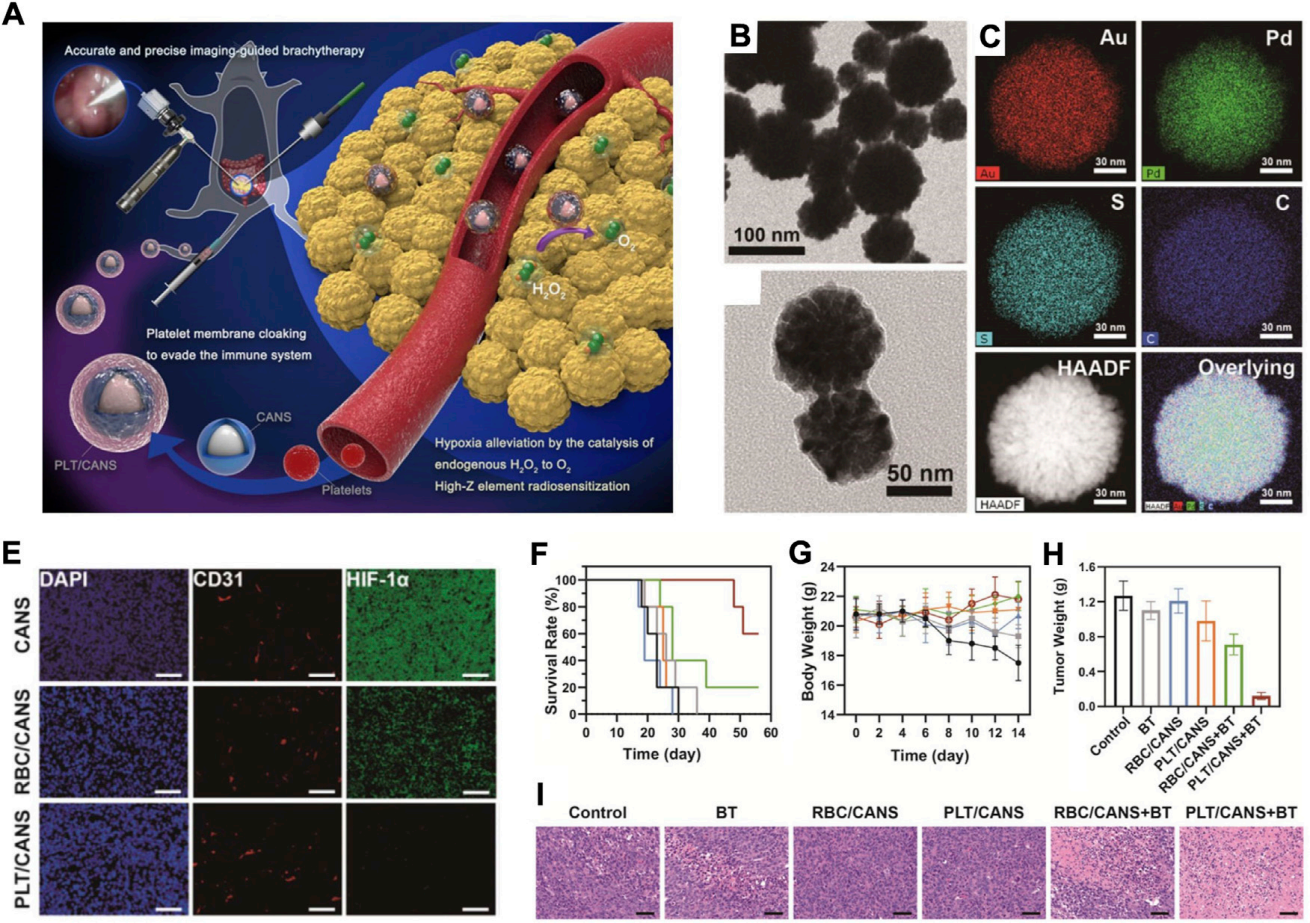
**(A)** The scheme showing the preparation of PLT/CANS and its application in alleviating tumor hypoxia. **(B)** TEM image of CANS. **(C,D)** TEM image **(C)** and **(D)** element mapping image of PLT/CANS. **(E)** Immunofluorescence analysis of tumor sections stained with TUNEL and HIF-1α after indicated treatments (scale bars: 50 μm). **(F–H)** Survival rates **(F)**; body weights **(G)** and tumor masses **(H)** of mice after indicated treatments. **(I)** H&E staining of tumor slices collected from different groups of mice (scale bars: 50 μm). Reprocessed with permission from ref 62. Copyright 2021, Ivyspring International Publisher.

In addition to RBC membranes, platelet membranes have also been utilized to design biomimetic nanomaterials (Hu et al., 2015a; Hu et al., 2015b). These biomimetic nanomaterials retained the surface glycoproteins of platelets as well as the tumour active targeting function (Sarkar et al., 2013; Zhang et al., 2020). In addition, these biomimetic nanomaterials could be activated by tumour cells, leading to size tuning, deep tumour penetration, and better antitumor output (Zuo et al., 2018). In a recent study, Bao and his coworkers fabricated a platelet membrane-based biomimetic nanomaterial by coating core-shell Au@AuPd nanospheres with a platelet membrane (PLT/CANS) for enhanced interstitial brachytherapy (BT) (Figure 6A) (Lyu et al., 2021). TEM and element mapping results showed that the as-prepared CANS had a core-shell spherical structure (Figures 6B–D). Compared to RBC membrane-coated nanoplatforms, PLT-derived membranes not only can effectively evade blood clearance but also have a special tumour targeting ability. From the immunofluorescence analysis of tumour sections stained with TUNEL and HIF-1α, one can see that PLT/CANS treatment could conquer tumour hypoxia (Figure 6E). After the combinational treatment of PLT/CANS and BT, the tumour-bearing mice showed longer survival, stable bodyweight growth, and lower tumour mass (Figures 6F–H). Histological analysis further showed that the combinational treatment of PLT/CANS and BT most effectively induced cellular apoptosis (Figure 6I). Overall, this work showed that platelet membrane-based biomimetic nanomaterials could be a robust and efficient strategy for tumour treatment.

### Cancer cell membrane-based biomimetic nanomaterials

**FIGURE 7:**
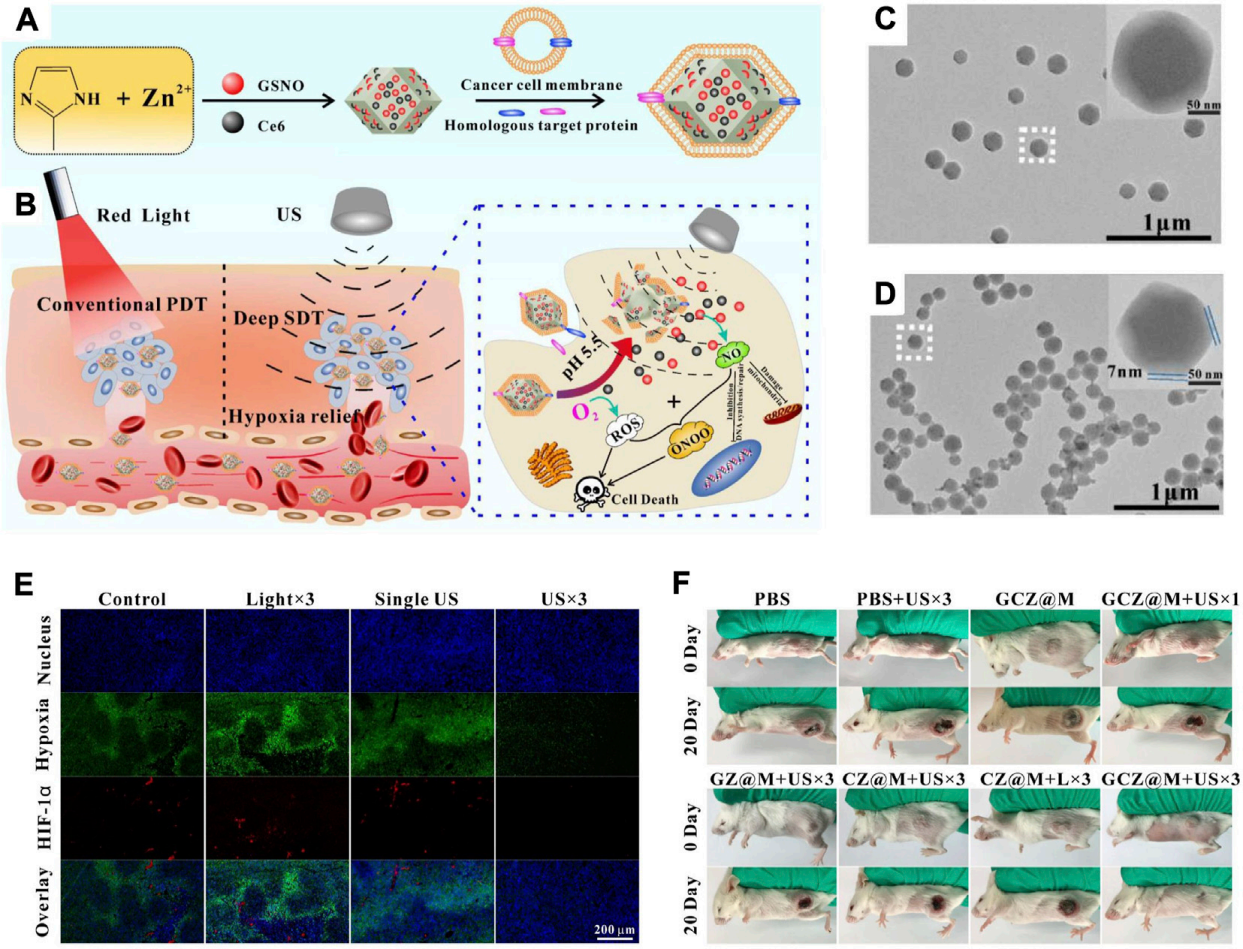
**(A)** Schematic illustration of the synthesis process of GCZ@M. **(B)** Schematic illustration of the tumor therapy principle. **(C,D)** TEM and magnification images (inset) of GCZ **(C)** and GCZ@M **(D)**. **(E)** Immunofluorescence analysis of tumor sections after indicated treatments. **(F)** Photos of tumor-bearing mice after indicated treatments. Reprocessed with permission from ref 71. Copyright 2019, Elsevier Ltd. All rights reserved.

As the study develops in depth, it has been found that the tumour cell membrane has a selective targeting homing ability due to the self-recognition to oncogenic cell lines. As a cancer cell membrane with a negative charge, the positive nanoparticles are easily bound to the tumour cell membrane, resulting in preferential uptake in tumors (Zhang et al., 2019b; Fang et al., 2021). Zhao et al. developed a biomimetic nanoplatform based on homologous tumour cell membranes with homologous targeting and low phototoxicity (Figure 7A) (An et al., 2020). Cancer cell membrane-based biomimetic nanomaterials (GCZ@M) were formed by the encapsulation of GSNO/Ce_6_@ZIF-8 by the cancer cell membrane. The *in vivo* results revealed that GCZ@M could accumulate in tumours with the help of the homologous tumour cell membrane. The encapsulated drug that accumulates in tumours could be sustainably released, triggered by pH and ultrasound. Then, the therapeutic effect of sonodynamic therapy could be improved by the effect of overcoming hypoxia in tumours through the use of ultrasound and the therapeutic properties of GCZ (Figure 7B). As tumours have a dense stroma, GCZ@M showed a uniform size and good dispersion, while the cancer cell membrane was a thin film, which did not reduce the inherent size advantage of the nanoparticles (Figures 7C,D)**.** Immunofluorescence analysis of tumour sections after GCZ@M treatment revealed that the NO and ROS produced by US could relieve tumour hypoxia, while the photographs of tumour-bearing mice after GCZ@M treatment exhibited an excellent therapeutic outcome (Figures 7E,F).

## Catalase-facilitated tumour oxygenation strategies

Hydrogen peroxide (H_2_O_2_) is overexpressed in the TME (Han et al., 2019; Li et al., 2020b). The overexpressed H_2_O_2_ can be catalysed into O_2_ by the natural catalase (CAT) or CAT-like nanozymes (Wang et al., 2020). The *in situ* generated O_2_ in tumour sites can be taken advantage of to overcome hypoxia-confined poor tumour treatment outcomes.

### Catalase -based biomimetic nanomaterials

**FIGURE 8:**
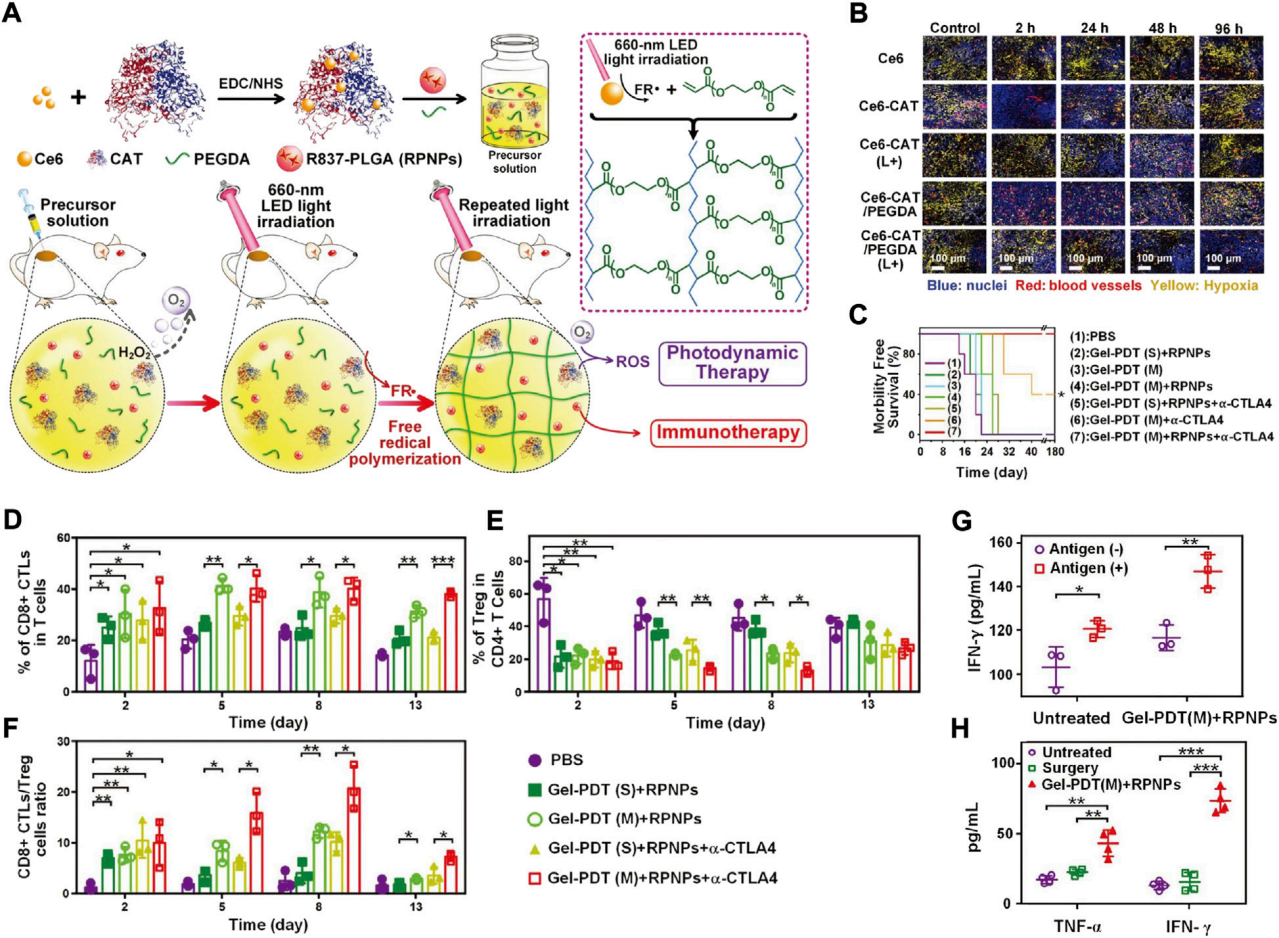
**(A)** Schematic illustration of the light-triggered fabrication of Ce6-CAT/PEGDA hydrogel in tumor sites. **(B)** Immunofluorescence analysis of tumor sections stained with anti-pimonidazole antibody (yellow) and anti-CD31 antibody (red). TUNEL **(C)** Morbidity free survival of the tumor-bearing mice after indicated treatments (*n* = 7). **(D–F)** Proportions of tumor-infiltrating CD8^+^ CTLs **(D)**; Treg cells **(E)** and CD8^+^ CTLs/Treg cells **(F)** after indicated treatments (*n* = 3). **(G)** The secretion of IFN-γ from restimulated splenocytes by the addition of the dead cell (antigen) *in vitro* (*n* = 3). **(H)** Serous cytokine (TNF-ɑ, IFN-γ) levels after indicated treatments (*n* = 4). Statistical analysis was performed *via* one-way ANOVA using the Tukey post-test. *p* < 0.05; **, *p* < 0.01; ***, *p* < 0.001. Reprocessed with permission from ref 78. Copyright 2019 WILEY-VCH Verlag GmbH & Co. KGaA, Weinheim.

As a natural antioxidant enzyme, CAT suffers from limited retention in tumour sites, leading to the limited generation of O_2_ in deep hypoxic areas (Ansar et al., 2020; Li et al., 2020c). To address this problem, a series of CAT-based biomimetic nanomaterials was developed (Yen et al., 2019). Meng *et al* reported Ce_6_-CAT/RPNPs/PEGDA biomimetic nanomaterials (Meng et al., 2019). As a light-induced *in situ* hybrid hydrogel system, Ce_6_-CAT/RPNPs/PEGDA realized sustained tumour hypoxia modulation to facilitate both PDT and immunotherapy (Figure 8A). Immunofluorescence staining with pimonidazole as the hypoxyprobe was conducted to track the tumour hypoxia states. While the decomposition of H_2_O_2_ by CAT could generate O_2_ in the TME, the poor retention ability of Ce_6_-CAT alleviated the tumour hypoxia status at the initial state, but it was recovered within 48 h after PDT treatment. In contrast, after light irradiation, the Ce_6_-CAT/PEGDA group showed a striking reduction in the degree of hypoxia over a period of 96 h, indicating that the retention of CAT could realize persistent tumour hypoxia relief (Figure 8B). With the sustained modulation of the hypoxic TME as well as the additional engagement of α-CTLA4 checkpoint blockade, the survival time was significantly prolonged (Figure 8C). Additionally, after combinational treatment with Ce_6_-CAT/RPNPs/PEGDA and α-CTLA4 checkpoint blockade, the antitumor immunotherapeutic responses were also strengthened (Figures 8D–F), as reflected by the increased populations of CD8^+^ CTLs (cytotoxic T lymphocytes) and the decreased populations of Treg cells (a kind of immunosuppressive cell). TNF-α and IFN-γ, two major indicators related to antitumor immune responses, were also detected in sera after different treatments. The TNF-α and IFN-γ concentrations were both significantly increased in the RPNPs/Ce_6_-CAT/PEGDA group, which is useful for effectively preventing tumour recurrence (Figures 8G,H).

### Catalase -like nanozyme-based biomimetic nanomaterials

**FIGURE 9:**
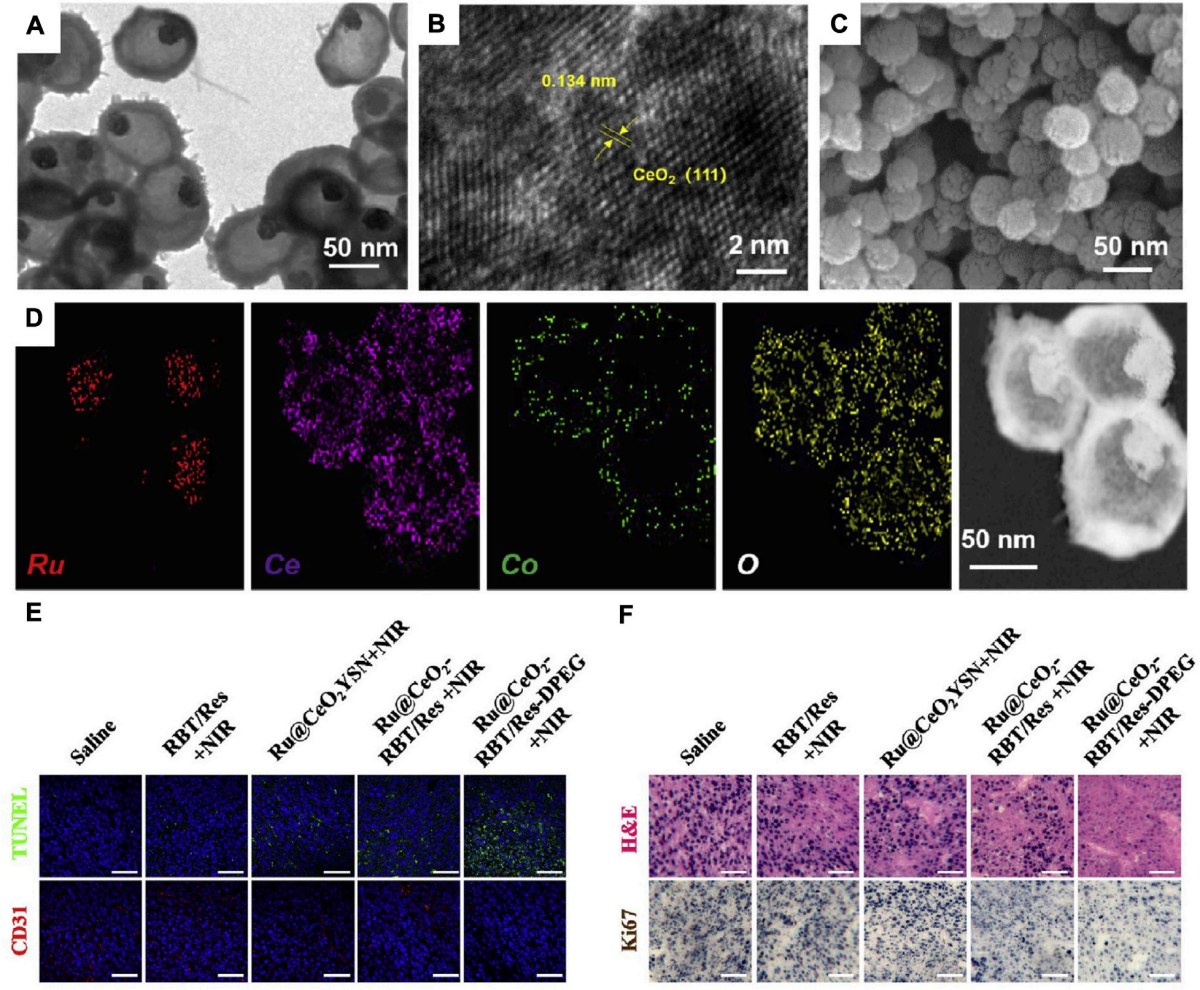
**(A–D)** TEM images **(A)**; HTEM images **(B)**; SEM images **(C)** and STEM-EDX elemental mapping images of Ru@CeO_2_ YSNs. **(E)** Immunofluorescence analysis of tumor sections stained with TUNEL and CD31. **(F)** H&E and Ki-67co-staining of tumor slices collected from different groups of mice. Reprocessed with permission from ref 81. Copyright 2020 Elsevier Ltd. All rights reserved.

CAT-like nanozymes are nanomaterials that possess CAT-like catalytic activity and enable the decomposition of H_2_O_2_ into O_2_ (Gordijo et al., 2015; Lin et al., 2018). Recently, Qin and coworkers tailor made hollow Ru@CeO_2_ (a type of CAT-like nanozyme)-based biomimetic nanomaterial (Ru@CeO_2_-RBT/Res-DPEG) to alleviate tumour hypoxia and inhibit tumour metastasis and recurrence (Zhu et al., 2020). TEM images showed that the as-obtained Ru@CeO_2_ has a uniform spherical core-shell structure with an average size of ∼70 nm (Figure 9A). Meanwhile, the high-resolution TEM images showed that the lattice fringes with a spacing of 0.314 nm perfectly matched the (111) plane of CeO_2_, indicating that the 3-6 nm interpenetrating CeO_2_ nanocrystals composed the mesoporous shell of Ru@CeO_2_ (Figure 9B). In addition, SEM images showed that Ru@CeO_2_ had a roughly spherical morphology (Figure 9C), and STEM-EDX elemental mapping confirmed that Ru (red) was the core component of Ru@CeO_2_, while Ce (purple) and O (yellow) were the shell components of Ru@CeO_2_ (Figure 9D). Subsequently, Ru@CeO_2_ was coated with RBT/Res-DPEG to improve the biocompatibility, reduce protein adsorption, and prolong the blood circulation time. Since Ru@CeO_2_-RBT/Res-DPEG has the ability to effectively deplete H_2_O_2_ in the TME to generate O_2_ and alleviate tumour hypoxia, Ru@CeO_2_-RBT/Res-DPEG exerted a dramatic antitumor effect, as verified by more than half area of cell apoptosis (Figure 9E), serious cell necrosis and inhibited cell proliferative activity (Figure 9F).

## Conclusion and outlook

Herein, we have summarized the latest progress in biomimetic nanomaterial-driven tumour oxygenation strategies. We depicted in detail the multiple merits of these biomimetic nanomaterials, including immune evasion, prolongation of the circulation time, enhanced biocompatibility, and wonderful tumour-targeting and tumour-selecting efficacy. In light of these multiple merits, the biomimetic nanomaterials exerted superior effects on alleviating the hypoxic TME and improving tumour treatment outputs relative to other materials (Li et al., 2021b; Gowda et al., 2022; Li et al., 2022).

Although the current biomimetic nanomaterial-driven tumour oxygenation strategies have remarkably surmounted hypoxia-associated resistance in tumour treatment, there are still some ongoing challenges that should be solved before these strategies can be applied to clinical cancer treatment.

(1) In human endogenous protein-facilitated tumour oxygenation strategies, the protein structure may be altered due to covalent or noncovalent modifications and the application of organic solvents. Additionally, the release of the delivery systems from the human endogenous protein is also a challenge (Yang et al., 2020).
(2) In red blood cell-facilitated tumour oxygenation strategies, the limited proliferative ability of red blood cells extremely confines the mass production of red blood cell-based biomimetic nanomaterials and thus cannot meet clinical needs (Tang et al., 2016).
(3) In cell membrane-facilitated tumour oxygenation strategies, the cell membrane has difficulty completely covering the surface of nanomaterials and is easily detached from the surface of nanomaterials due to the lack of covalent bond constraints, thus limiting clinical applications (Liu et al., 2021b).
(4) In catalase-facilitated tumour oxygenation strategies, the intratumourally endogenous H_2_O_2_ amount is limited, which greatly restricts the ability of biomimetic nanomaterials to generate sufficient *in situ* O_2_ (Song et al., 2018).

We envision that the ongoing challenges will be solved in the future and that biomimetic nanomaterial-empowered tumour oxygenation strategies will promote the development of cancer treatment in the clinic.

## References

[B1] Ai X. Z. Hu M. Wang Z. M. Lyu L. N. Zhang W. M. Li J. (2018). Enhanced cellular ablation by attenuating hypoxia status and reprogramming tumor-associated macrophages via NIR light-responsive upconversion nanocrystals. Bioconjug. Chem. 29 (4), 928–938. 10.1021/acs.bioconjchem.8b00068 29466856

[B2] An J. Hu Y. G. Li C. Hou X. L. Cheng K. Zhang B. (2020). A pH/Ultrasound dual-response biomimetic nanoplatform for nitric oxide gas-sonodynamic combined therapy and repeated ultrasound for relieving hypoxia. Biomaterials 230, 119636. 10.1016/j.biomaterials.2019.119636 31785776

[B3] Ansar M. Ivanciuc T. Garofalo R. P. Casola A. (2020). Increased lung catalase activity confers protection against experimental RSV infection. Sci. Rep. 10 (1), 3653. 10.1038/s41598-020-60443-2 32107411 PMC7046725

[B4] Ayob A. Z. Ramasamy T. S. (2021). Prolonged hypoxia switched on cancer stem cell-like plasticity in HepG2 tumourspheres cultured in serum-free media. Vitro Cell. Dev. Biol. -Animal. 57 (9), 896–911. 10.1007/s11626-021-00625-y 34750738

[B5] Baggiani A. Ierardi A. Caspani B. Motta F. Toniolo D. Belloni P. (2011). Hypoxic liver perfusion with mitomycin-C for treating multifocal metastases and unresectable primary tumours: A single-centre series of 42 patients. Radiol. Med. 116 (8), 1239–1249. 10.1007/s11547-011-0724-3 21892710

[B6] Baumann R. Depping R. Delaperriere M. Dunst J. (2016). Targeting hypoxia to overcome radiation resistance in head & neck cancers: Real challenge or clinical fairytale? Expert Rev. Anticancer Ther. 16 (7), 751–758. 10.1080/14737140.2016.1192467 27253509

[B7] Beutler E. Dale G. L. Guinto D. E. Kuhl W. (1977). Enzyme replacement therapy in gaucher's disease: Preliminary clinical trial of a new enzyme preparation. Proc. Natl. Acad. Sci. U. S. A. 74 (10), 4620–4623. 10.1073/pnas.74.10.4620 200923 PMC431998

[B8] Bouleftour W. Rowinski E. Louati S. Sotton S. Wozny A. S. Moreno-Acosta P. (2021). A review of the role of hypoxia in radioresistance in cancer therapy. Med. Sci. Monit. 27, e934116. 10.12659/msm.934116 34728593 PMC8573967

[B9] Brahimi-Horn M. C. Chiche J. Pouyssegur J. (2007). Hypoxia and cancer. J. Mol. Med. 85 (12), 1301–1307. 10.1007/s00109-007-0281-3 18026916

[B10] Carlson D. J. Keall P. J. Loo B. W. Chen Z. J. Brown J. M. (2011). Hypofractionation results in reduced tumor cell kill compared to conventional fractionation for tumors with regions of hypoxia. Int. J. Radiat. Oncology*Biology*Physics 79 (4), 1188–1195. 10.1016/j.ijrobp.2010.10.007 PMC3053128 21183291

[B11] Carvalho T. M. A. Di Molfetta D. Greco M. R. Koltai T. Alfarouk K. O. Reshkin S. J. (2021). Tumor microenvironment features and chemoresistance in pancreatic ductal adenocarcinoma: Insights into targeting physicochemical barriers and metabolism as therapeutic approaches. Cancers 13 (23), 6135. 10.3390/cancers13236135 34885243 PMC8657427

[B12] Chang M. Y. Wang M. Wang M. F. Shu M. M. Ding B. B. Li C. X. (2019). A multifunctional cascade bioreactor based on hollow-structured Cu2MoS4 for synergetic cancer chemo-dynamic therapy/starvation therapy/phototherapy/immunotherapy with remarkably enhanced efficacy. Adv. Mater 31 (51), 1905271. 10.1002/adma.201905271 31680346

[B13] Chen K. Li H. Xu Y. Ge H. Ning X. (2022). Photoactive "bionic virus" robustly elicits the synergy anticancer activity of immunophotodynamic therapy. ACS Appl. Mat. Interfaces 14 (3), 4456–4468. 10.1021/acsami.1c23983 35021012

[B14] Chen Z. Wang Z. Gu Z. (2019). Bioinspired and biomimetic nanomedicines. Acc. Chem. Res. 52 (5), 1255–1264. 10.1021/acs.accounts.9b00079 30977635 PMC7293770

[B15] Chu C. C. Lin H. R. Liu H. Wang X. Y. Wang J. Q. Zhang P. F. (2017). Tumor microenvironment-triggered supramolecular system as an *in situ* nanotheranostic generator for cancer phototherapy. Adv. Mat. 29 (23). 10.1002/adma.201605928 PMC5499384 28417485

[B16] Delprat V. Tellier C. Demazy C. Raes M. Feron O. Michiels C. (2020). Cycling hypoxia promotes a pro-inflammatory phenotype in macrophages via JNK/p65 signaling pathway. Sci. Rep. 10 (1), 882. 10.1038/s41598-020-57677-5 31964911 PMC6972721

[B17] Ding Y. Wang Y. Zhou J. Gu X. Wang W. Liu C. (2014). Direct cytosolic siRNA delivery by reconstituted high density lipoprotein for target-specific therapy of tumor angiogenesis. Biomaterials 35 (25), 7214–7227. 10.1016/j.biomaterials.2014.05.009 24875759

[B18] Dong S. Dong Y. Jia T. Liu S. Liu J. Yang D. (2020). GSH-depleted nanozymes with hyperthermia-enhanced dual enzyme-mimic activities for tumor nanocatalytic therapy. Adv. Mat. 32 (42), e2002439. 10.1002/adma.202002439 32914495

[B19] Fang H. Gai Y. Wang S. Liu Q. Zhang X. Ye M. (2021). Biomimetic oxygen delivery nanoparticles for enhancing photodynamic therapy in triple-negative breast cancer. J. Nanobiotechnology 19 (1), 81. 10.1186/s12951-021-00827-2 33743740 PMC7981819

[B20] Feng Q. Yang X. Hao Y. Wang N. Feng X. Hou L. (2019). Cancer cell membrane-biomimetic nanoplatform for enhanced sonodynamic therapy on breast cancer via autophagy regulation strategy. ACS Appl. Mat. Interfaces 11 (36), 32729. 10.1021/acsami.9b10948 31415145

[B21] Gabrilovich D. I. Chen H. L. Girgis K. R. Cunningham H. T. Meny G. M. Nadaf S. (1996). Production of vascular endothelial growth factor by human tumors inhibits the functional maturation of dendritic cells. Nat. Med. 2 (10), 1096–1103. 10.1038/nm1096-1096 8837607

[B22] Gao S. Zheng P. Li Z. Feng X. Yan W. Chen S. (2018). Biomimetic O2-Evolving metal-organic framework nanoplatform for highly efficient photodynamic therapy against hypoxic tumor. Biomaterials 178, 83–94. 10.1016/j.biomaterials.2018.06.007 29913389

[B23] Gao Z. Li Y. Zhang Y. Cheng K. An P. Chen F. (2020). Biomimetic platinum nanozyme immobilized on 2D metal-organic frameworks for mitochondrion-targeting and oxygen self-supply photodynamic therapy. ACS Appl. Mat. Interfaces 12 (2), 1963–1972. 10.1021/acsami.9b14958 31873002

[B24] Gong C. Yu X. Zhang W. Han L. Wang R. Wang Y. (2021). Regulating the immunosuppressive tumor microenvironment to enhance breast cancer immunotherapy using pH-responsive hybrid membrane-coated nanoparticles. J. Nanobiotechnology 19 (1), 58. 10.1186/s12951-021-00805-8 33632231 PMC7905864

[B25] Gong L. Zhang Y. Zhao J. Zhang Y. Tu K. Jiao L. (2022). All-in-one biomimetic nanoplatform based on hollow polydopamine nanoparticles for synergistically enhanced radiotherapy of colon cancer. Small 18 (14), e2107656. 10.1002/smll.202107656 35150039

[B26] Gordijo C. R. Abbasi A. Z. Amini M. A. Lip H. Y. Maeda A. Cai P. (2015). Design of hybrid MnO2-polymer-lipid nanoparticles with tunable oxygen generation rates and tumor accumulation for cancer treatment. Adv. Funct. Mater 25 (12), 1858–1872. 10.1002/adfm.201404511

[B27] Gowda V. K. Rosén T. Roth S. V. Söderberg L. D. Lundell F. (2022). Nanofibril alignment during assembly revealed by an X-ray scattering-based digital twin. ACS Nano 16 (2), 2120–2132. 10.1021/acsnano.1c07769 35104107 PMC8867913

[B28] Han Y. K. Park G. Y. Bae M. J. Kim J. S. Jo W. S. Lee C. G. (2019). Hypoxia induces immunogenic cell death of cancer cells by enhancing the exposure of cell surface calreticulin in an endoplasmic reticulum stress-dependent manner. Oncol. Lett. 18 (6), 6269–6274. 10.3892/ol.2019.10986 31788104 PMC6865837

[B29] Hu C. M. Fang R. H. Wang K. C. Luk B. T. Thamphiwatana S. Dehaini D. (2015). Nanoparticle biointerfacing by platelet membrane cloaking. Nature 526 (7571), 118–121. 10.1038/nature15373 26374997 PMC4871317

[B30] Hu Q. Sun W. Qian C. Wang C. Bomba H. N. Gu Z. (2015). Anticancer platelet-mimicking nanovehicles. Adv. Mater 27 (44), 7043–7050. 10.1002/adma.201503323 26416431 PMC4998740

[B31] Huang H. Zhang C. Wang X. L. Shao J. S. Chen C. Li H. M. (2020). Overcoming hypoxia-restrained radiotherapy using an erythrocyte-inspired and glucose-activatable platform. Nano Lett. 20 (6), 4211–4219. 10.1021/acs.nanolett.0c00650 32352796

[B32] Huang Y. Yuan J. Righi E. Kamoun W. S. Ancukiewicz M. Nezivar J. (2012). Vascular normalizing doses of antiangiogenic treatment reprogram the immunosuppressive tumor microenvironment and enhance immunotherapy. Proc. Natl. Acad. Sci. U. S. A. 109 (43), 17561. 10.1073/pnas.1215397109 23045683 PMC3491458

[B33] Huo D. Jiang X. Q. Hu Y. (2020). Recent advances in nanostrategies capable of overcoming biological barriers for tumor management. Adv. Mat. 32 (27), 1904337. 10.1002/adma.201904337 31663198

[B34] Jiang T. Heng S. Huang X. Zheng L. Kai D. Loh X. J. (2019). Biomimetic poly(poly(ε-caprolactone)-polytetrahydrofuran urethane) based nanofibers enhanced chondrogenic differentiation and cartilage regeneration. J. Biomed. Nanotechnol. 15 (5), 1005–1017. 10.1166/jbn.2019.2748 30890231

[B35] Jiang W. Zhang Z. Wang Q. Dou J. X. Zhao Y. Y. Ma Y. C. (2019). Tumor reoxygenation and blood perfusion enhanced photodynamic therapy using ultrathin graphdiyne oxide nanosheets. Nano Lett. 19 (6), 4060–4067. 10.1021/acs.nanolett.9b01458 31136712

[B36] Kuo C. T. Chiang C. L. Chang C. H. Liu H. K. Huang G. S. Huang R. Y. (2014). Modeling of cancer metastasis and drug resistance via biomimetic nano-cilia and microfluidics. Biomaterials 35 (5), 1562–1571. 10.1016/j.biomaterials.2013.11.008 24269156

[B37] Lee J. B. Kim D. H. Yoon J. K. Park D. B. Kim H. S. Shin Y. M. (2020). Microchannel network hydrogel induced ischemic blood perfusion connection. Nat. Commun. 11 (1), 615. 10.1038/s41467-020-14480-0 32001693 PMC6992688

[B38] Li C. Yang X. Q. An J. Cheng K. Hou X. L. Zhang X. S. (2020). Red blood cell membrane-enveloped O2 self-supplementing biomimetic nanoparticles for tumor imaging-guided enhanced sonodynamic therapy. Theranostics 10 (2), 867–879. 10.7150/thno.37930 31903156 PMC6929970

[B39] Li L. H. Shih Y. L. Huang J. Y. Wu C. J. Huang Y. W. Huang H. H. (2020). Protection from hydrogen peroxide stress relies mainly on AhpCF and KatA2 in Stenotrophomonas maltophilia. J. Biomed. Sci. 27 (1), 37. 10.1186/s12929-020-00631-4 32093695 PMC7041247

[B40] Li M. Yin F. Song L. Mao X. Li F. Fan C. (2021). Nucleic acid tests for clinical translation. Chem. Rev. 121 (17), 10469. 10.1021/acs.chemrev.1c00241 34254782

[B41] Li Q. Lin B. Li Y. Lu N. (2021). Erythrocyte-camouflaged mesoporous titanium dioxide nanoplatform for an ultrasound-mediated sequential therapies of breast cancer. Int. J. Nanomedicine 16, 3875–3887. 10.2147/ijn.s301855 34135582 PMC8197575

[B42] Li W. Wang C. Wang Z. Gou L. Zhou Y. Peng G. (2022). Physically cross-linked DNA hydrogel-based sustained cytokine delivery for *in situ* diabetic alveolar bone rebuilding. ACS Appl. Mat. Interfaces 14 (22), 25173–25182. 10.1021/acsami.2c04769 35638566

[B43] Li X. Feng X. Q. Sun C. S. Liu Y. X. Zhao Q. F. Wang S. L. (2020). Mesoporous carbon-manganese nanocomposite for multiple imaging guided oxygen-elevated synergetic therapy. J. Control. Release 319, 104–118. 10.1016/j.jconrel.2019.12.042 31881317

[B44] Li X. Gu G. Soliman F. Sanders A. J. Wang X. Liu C. (2018). The evaluation of durative transfusion of endostar combined with chemotherapy in patients with advanced non-small cell lung cancer. Chemotherapy 63 (4), 214–219. 10.1159/000493098 30347389

[B45] Liang H. Wu Y. Ou X. Y. Li J. Y. Li J. (2017). Au@Pt nanoparticles as catalase mimics to attenuate tumor hypoxia and enhance immune cell-mediated cytotoxicity. Nanotechnology 28 (46), 465702. 10.1088/1361-6528/aa8d9c 28925921

[B46] Liang R. J. Liu L. L. He H. M. Chen Z. K. Han Z. Q. Luo Z. Y. (2018). Oxygen-boosted immunogenic photodynamic therapy with gold nanocages@manganese dioxide to inhibit tumor growth and metastases. Biomaterials 177, 149–160. 10.1016/j.biomaterials.2018.05.051 29890364

[B47] Lin T. Zhao X. Zhao S. Yu H. Cao W. Chen W. (2018). O2-generating MnO2 nanoparticles for enhanced photodynamic therapy of bladder cancer by ameliorating hypoxia. Theranostics 8 (4), 990–1004. 10.7150/thno.22465 29463995 PMC5817106

[B48] Lindau D. Gielen P. Kroesen M. Wesseling P. Adema G. J. (2013). The immunosuppressive tumour network: Myeloid-derived suppressor cells, regulatory T cells and natural killer T cells. Immunology 138 (2), 105–115. 10.1111/imm.12036 23216602 PMC3575763

[B49] Liu C. Z. Chen Y. X. Zhao J. Wang Y. Shao Y. L. Gu Z. N. (2021). Self-assembly of copper-DNAzyme nanohybrids for dual-catalytic tumor therapy. Angew. Chem. Int. Ed. Engl. 60 (26), 14445. 10.1002/ange.202101744 33822451

[B50] Liu J. Cabral H. Song B. Aoki I. Chen Z. Y. Nishiyama N. (2021). Nanoprobe-based magnetic resonance imaging of hypoxia predicts responses to radiotherapy, immunotherapy, and sensitizing treatments in pancreatic tumors. ACS Nano 15 (8), 13526. 10.1021/acsnano.1c04263 34355882

[B51] Liu Y. L. Pan Y. X. Cao W. Xia F. F. Liu B. Niu J. Q. (2019). A tumor microenvironment responsive biodegradable CaCO3/MnO2- based nanoplatform for the enhanced photodynamic therapy and improved PD-L1 immunotherapy. Theranostics 9 (23), 6867–6884. 10.7150/thno.37586 31660074 PMC6815945

[B52] Lyu M. Chen M. Liu L. Zhu D. Wu X. Li Y. (2021). A platelet-mimicking theranostic platform for cancer interstitial brachytherapy. Theranostics 11 (15), 7589–7599. 10.7150/thno.61259 34158868 PMC8210607

[B53] Meng L. Wang C. Lu Y. Sheng G. Yang L. Wu Z. (2021). Targeted regulation of blood-brain barrier for enhanced therapeutic efficiency of hypoxia-modifier nanoparticles and immune checkpoint blockade antibodies for glioblastoma. ACS Appl. Mat. Interfaces 13 (10), 11657. 10.1021/acsami.1c00347 33684289

[B54] Meng Z. Q. Zhou X. F. Xu J. Han X. Dong Z. L. Wang H. R. (2019). Light-triggered *in situ* gelation to enable robust photodynamic-immunotherapy by repeated stimulations. Adv. Mater 31 (24), 1900927. 10.1002/adma.201900927 31012164

[B55] Noman M. Z. Desantis G. Janji B. Hasmim M. Karray S. Dessen P. (2014). PD-L1 is a novel direct target of HIF-1α, and its blockade under hypoxia enhanced MDSC-mediated T cell activation. J. Exp. Med. 211 (5), 781–790. 10.1084/jem.20131916 24778419 PMC4010891

[B56] Sarkar S. Alam M. A. Shaw J. Dasgupta A. K. (2013). Drug delivery using platelet cancer cell interaction. Pharm. Res. 30 (11), 2785–2794. 10.1007/s11095-013-1097-1 23739991

[B57] Shi X. Yang W. Ma Q. Lu Y. Xu Y. Bian K. (2020). Hemoglobin-mediated biomimetic synthesis of paramagnetic O2-evolving theranostic nanoprobes for MR imaging-guided enhanced photodynamic therapy of tumor. Theranostics 10 (25), 11607. 10.7150/thno.46228 33052236 PMC7545996

[B58] Song C. H. Tang C. C. Xu W. G. Ran J. C. Wei Z. Wang Y. F. (2020). Hypoxia-targeting multifunctional nanoparticles for sensitized chemotherapy and phototherapy in head and neck squamous cell carcinoma. Int. J. Nanomedicine 15, 347–361. 10.2147/ijn.s233294 32021184 PMC6980849

[B59] Song X. J. Xu J. Liang C. Chao Y. Jin Q. T. Wang C. (2018). Self-supplied tumor oxygenation through separated liposomal delivery of H2O2 and catalase for enhanced radio-immunotherapy of cancer. Nano Lett. 18 (10), 6360–6368. 10.1021/acs.nanolett.8b02720 30247918

[B60] Tang W. Zhen Z. Wang M. Wang H. Chuang Y. J. Zhang W. (2016). Red blood cell-facilitated photodynamic therapy for cancer treatment. Adv. Funct. Mat. 26 (11), 1757–1768. 10.1002/adfm.201504803 PMC6867707 31749670

[B61] Tang Y. Q. Chen T. F. Zhang Y. Zhao X. C. Zhang Y. Z. Wang G. Q. (2020). The tumor immune microenvironment transcriptomic subtypes of colorectal cancer for prognosis and development of precise immunotherapy. Gastroenterol. Rep. (Oxf) 8 (5), 381–389. 10.1093/gastro/goaa045 33163194 PMC7603874

[B62] Voron T. Colussi O. Marcheteau E. Pernot S. Nizard M. Pointet A. L. (2015). VEGF-A modulates expression of inhibitory checkpoints on CD8+ T cells in tumors. J. Exp. Med. 212 (2), 139–148. 10.1084/jem.20140559 25601652 PMC4322048

[B63] Wang D. Wu H. Phua S. Z. F. Yang G. Qi Lim W. Gu L. (2020). Self-assembled single-atom nanozyme for enhanced photodynamic therapy treatment of tumor. Nat. Commun. 11 (1), 357. 10.1038/s41467-019-14199-7 31953423 PMC6969186

[B64] Wang P. Wang X. Luo Q. Li Y. Lin X. Fan L. (2019). Fabrication of red blood cell-based multimodal theranostic probes for second near-infrared window fluorescence imaging-guided tumor surgery and photodynamic therapy. Theranostics 9 (2), 369–380. 10.7150/thno.29817 30809280 PMC6376196

[B65] Wu J. Y. Huang T. W. Hsieh Y. T. Wang Y. F. Yen C. C. Lee G. L. (2020). Cancer-derived succinate promotes macrophage polarization and cancer metastasis via succinate receptor. Mol. Cell 77 (2), 213–227.e5. 10.1016/j.molcel.2019.10.023 31735641

[B66] Yang T. Wang Y. Ke H. T. Wang Q. L. Lv X. Y. Wu H. (2016). Protein-nanoreactor-assisted synthesis of semiconductor nanocrystals for efficient cancer theranostics. Adv. Mater 28 (28), 5923–5930. 10.1002/adma.201506119 27165472

[B67] Yang W. T. Guo W. S. Chang J. Zhang B. B. (2017). Protein/peptide-templated biomimetic synthesis of inorganic nanoparticles for biomedical applications. J. Mat. Chem. B 5 (3), 401–417. 10.1039/c6tb02308h 32263655

[B68] Yang W. T. Guo W. S. Le W. J. Lv G. X. Zhang F. H. Shi L. (2016). Albumin-bioinspired Gd:CuS nanotheranostic agent for *in vivo* photoacoustic/magnetic resonance imaging-guided tumor-targeted photothermal therapy. ACS Nano 10 (11), 10245. 10.1021/acsnano.6b05760 27791364

[B69] Yang W. T. Wu X. L. Dou Y. Chang J. Xiang C. Y. Yu J. N. (2018). A human endogenous protein exerts multi-role biomimetic chemistry in synthesis of paramagnetic gold nanostructures for tumor bimodal imaging. Biomaterials 161, 256–269. 10.1016/j.biomaterials.2018.01.050 29425846

[B70] Yang Z. Chen H. Yang P. Shen X. Hu Y. Cheng Y. (2022). Nano-oxygenated hydrogels for locally and permeably hypoxia relieving to heal chronic wounds. Biomaterials 282, 121401. 10.1016/j.biomaterials.2022.121401 35121358

[B71] Yang Z. Deng W. Zhang X. An Y. Liu Y. Yao H. (2022). Opportunities and challenges of nanoparticles in digestive tumours as anti-angiogenic therapies. Front. Oncol. 11, 789330. 10.3389/fonc.2021.789330 35083147 PMC8784389

[B72] Yang Z. Du Y. Sun Q. Peng Y. Wang R. Zhou Y. (2020). Albumin-based nanotheranostic probe with hypoxia alleviating potentiates synchronous multimodal imaging and phototherapy for glioma. ACS Nano 14 (5), 6191–6212. 10.1021/acsnano.0c02249 32320600

[B73] Yen T. Y. Stephen Z. R. Lin G. Y. Mu Q. X. Jeon M. Untoro S. (2019). Catalase-functionalized iron oxide nanoparticles reverse hypoxia-induced chemotherapeutic resistance. Adv. Healthc. Mater 8 (20), 1900826. 10.1002/adhm.201900826 PMC6919328 31557421

[B74] Yu M. Duan X. H. Cai Y. J. Zhang F. Jiang S. Q. Han S. S. (2019). Multifunctional nanoregulator reshapes immune microenvironment and enhances immune memory for tumor immunotherapy. Adv. Sci. (Weinh) 6 (16), 1900037. 10.1002/advs.201900037 31453054 PMC6702652

[B75] Zhang B. B. Jin H. T. Li Y. Chen B. D. Liu S. Y. Shi D. L. (2012). Bioinspired synthesis of gadolinium-based hybrid nanoparticles as MRI blood pool contrast agents with high relaxivity. J. Mat. Chem. 22 (29), 14494. 10.1039/c2jm30629h

[B76] Zhang C. Xia D. L. Liu J. H. Huo D. Jiang X. Q. Hu Y. (2020). Bypassing the immunosuppression of myeloid-derived suppressor cells by reversing tumor hypoxia using a platelet-inspired platform. Adv. Funct. Mater 30 (22), 2000189. 10.1002/adfm.202000189

[B77] Zhang L. Wang Z. Zhang Y. Cao F. Dong K. Ren J. (2018). Erythrocyte membrane cloaked metal-organic framework nanoparticle as biomimetic nanoreactor for starvation-activated colon cancer therapy. ACS Nano 12 (10), 10201. 10.1021/acsnano.8b05200 30265804

[B78] Zhang L. Y. Han F. (2018). Protein coated gold nanoparticles as template for the directed synthesis of highly fluorescent gold nanoclusters. Nanotechnology 29 (16), 165702. 10.1088/1361-6528/aaae47 29424708

[B79] Zhang M. K. Ye J. J. Li C. X. Xia Y. Wang Z. Y. Feng J. (2019). Cytomembrane-mediated transport of metal ions with biological specificity. Adv. Sci. (Weinh) 6 (17), 1900835. 10.1002/advs.201900835 31508286 PMC6724363

[B80] Zhang M. Ye J. J. Xia Y. Wang Z. Y. Li C. X. Wang X. S. (2019). Platelet-Mimicking biotaxis targeting vasculature-disrupted tumors for cascade amplification of hypoxia-sensitive therapy. ACS Nano 13 (12), 14230–14240. 10.1021/acsnano.9b07330 31714733

[B81] Zhao P. F. Zheng M. B. Luo Z. Y. Fan X. J. Sheng Z. H. Gong P. (2016). Oxygen nanocarrier for combined cancer therapy: Oxygen-boosted ATP-responsive chemotherapy with amplified ROS lethality. Adv. Healthc. Mat. 5 (17), 2161–2167. 10.1002/adhm.201600121 27253453

[B82] Zhao Y. Peng J. Li J. J. Huang L. Yang J. Y. Huang K. (2017). Tumor-targeted and clearable human protein-based MRI nanoprobes. Nano Lett. 17 (7), 4096–4100. 10.1021/acs.nanolett.7b00828 28581764

[B83] Zhou Z. G. Zhang B. L. Wang S. S. Zai W. J. Yuan A. Hu Y. Q. (2018). Perfluorocarbon nanoparticles mediated platelet blocking disrupt vascular barriers to improve the efficacy of oxygen-sensitive antitumor drugs. Small 14 (45), 1801694. 10.1002/smll.201801694 30307696

[B84] Zhu C. Q. Guo X. M. Luo L. H. Wu Z. Luo Z. Y. Jiang M. (2019). Extremely effective chemoradiotherapy by inducing immunogenic cell death and radio-triggered drug release under hypoxia alleviation. ACS Appl. Mat. Interfaces 11 (50), 46536. 10.1021/acsami.9b16837 31751119

[B85] Zhu X. F. Gong Y. C. Liu Y. A. Yang C. H. Wu S. J. Yuan G. L. (2020). Ru@CeO2 yolk shell nanozymes: Oxygen supply *in situ* enhanced dual chemotherapy combined with photothermal therapy for orthotopic/subcutaneous colorectal cancer. Biomaterials 242, 119923. 10.1016/j.biomaterials.2020.119923 32145506

[B86] Zuo H. Tao J. Shi H. He J. Zhou Z. Zhang C. (2018). Platelet-mimicking nanoparticles co-loaded with W18O49 and metformin alleviate tumor hypoxia for enhanced photodynamic therapy and photothermal therapy. Acta Biomater. 80, 296–307. 10.1016/j.actbio.2018.09.017 30223092

